# HDAC8 Deacetylates HIF-1α and Enhances Its Protein Stability to Promote Tumor Growth and Migration in Melanoma

**DOI:** 10.3390/cancers15041123

**Published:** 2023-02-09

**Authors:** Ji Yoon Kim, Hayoung Cho, Jung Yoo, Go Woon Kim, Yu Hyun Jeon, Sang Wu Lee, So Hee Kwon

**Affiliations:** College of Pharmacy Yonsei Institute of Pharmaceutical Sciences, Yonsei University, Incheon 21983, Republic of Korea

**Keywords:** epigenetics, HDAC8, HIF-1α, hypoxia, PCI-34051, melanoma

## Abstract

**Simple Summary:**

Melanoma is the deadliest form of skin cancer, with its incidence increasing significantly over the past few decades. The hypoxic microenvironment of melanoma gives rise to the expression of hypoxia-inducible factor-1 (HIF-1), which mediates various signaling pathways that lead to melanoma progression. In this study, we aim to identify an epigenetic regulator of HIF-1α that could be targeted for therapeutic interventions in melanoma. Here, we demonstrate that histone deacetylase 8 (HDAC8) deacetylates HIF-1α and enhances its stability. Accordingly, the pharmacological inhibition or the genetic ablation of HDAC8 suppresses the transcriptional activity of HIF-1 and demonstrates anti-cancer effects. Therefore, we suggest HDAC8 as a novel regulator of HIF-1α and a therapeutic target for melanoma treatment.

**Abstract:**

Melanoma is the most lethal type of skin cancer, and it causes more than 55,000 deaths annually. Although regional melanoma can be surgically removed, once melanoma metastasizes to other regions of the body, the survival rate drops dramatically. The current treatment options are chemotherapy, immunotherapy, and targeted therapy. However, the low response rate and the development of resistance necessitate the search for a novel therapeutic target in melanoma. Hypoxia-inducible factor-1 α (HIF-1α) is overexpressed in melanoma and plays a crucial role in driving malignant transformation in cancer cells. Here, we identified that histone deacetylase 8 (HDAC8) enhances the protein stability of HIF-1α. HDAC8 directly binds to and deacetylates HIF-1α, thereby promoting its protein stability. This, in turn, upregulates the transcriptional activity of HIF-1α and promotes the expressions of its target genes, such as hexokinase 2 (HK2) and glucose transporter 1 (GLUT1). The inhibition of HDAC8 suppresses the proliferation and metastasis of melanoma cells. Furthermore, *HDAC8* is correlated with *HIF1A* expression and poor prognosis in samples from patients with melanoma. These findings uncover a novel epigenetic mechanism that maintains HIF-1α stability and implicates the potential of HDAC8 inhibitors for melanoma therapy.

## 1. Introduction

Melanoma develops from melanocytes, which are highly differentiated cells that produce melanin, a macromolecule that protects against UV-induced damage [1]. Melanoma represents the most serious type of skin cancer, and it causes more than 55,000 deaths annually [2]. Worldwide, about 20 per 100,000 individuals are diagnosed with melanoma, and the incidence rates are still rapidly increasing [3]. According to the surveillance, epidemiology and end results (SEER), the 5-year survival rate (2011–2018) is 99.5% and 70.6% in localized and regional melanoma, respectively. However, once it metastasizes, the 5-year survival rate drops dramatically to 31.9% [4]. The current treatment strategies for metastatic melanoma include surgical excision, chemotherapy, immunotherapy, targeted therapy, photodynamic therapy, and radiotherapy [5]. In particular, the combination of BRAF and MEK inhibitors has been shown to be effective in patients with metastatic melanoma harboring BRAF mutations. So far, two BRAF inhibitors, vemurafenib and dabrafenib, have been approved by the Food and Drug Administration (FDA) [5]. Despite the advances made in melanoma therapy, adverse effects from accumulated toxicities and reduced efficiency from the development of resistance present barriers in the current melanoma treatment strategies. Thus, further investigation of the oncogenic pathway of melanoma and potential therapeutic targets are required.

Maintaining oxygen homeostasis is crucial for cell survival [6]. The depletion of O_2_ leads to increased levels of reactive oxygen species (ROS) that cause irreversible cellular damage [7,8]. Hypoxia-inducible factors (HIFs) function as master regulators of oxygen homeostasis and mediate adaptive responses to hypoxia to increase O_2_ availability. HIF-1 is a heterodimeric transcriptional activator that consists of a constitutively expressed β subunit and an α subunit. In normoxic conditions, the α subunit undergoes rapid proteasomal degradation. However, in hypoxic conditions, the hydroxylation of the prolyl residues in the α subunit is inhibited, allowing HIF-1α to escape proteasomal degradation and accumulate. This allows HIF-1α to promote the transcription of target genes by associating with the hypoxia-response element (HRE) [9]. Intratumoral hypoxia is a common feature in the tumor microenvironment due to its uncontrolled proliferating nature and the inadequate vascularization of cancer cells. Accumulating evidence shows that cancer cells exploit HIF-1-mediated pathways to promote tumor progression by activating the hallmarks of cancer, such as cell proliferation, glucose metabolism, metastasis, angiogenesis, and drug resistance [10]. Consistently, HIF-1α is overexpressed in multiple cancers, such as breast, colon, lung, and prostate carcinomas [11], and it has been recognized as an attractive target for anti-cancer therapy.

Epigenetics refers to the study of heritable changes in a chromosome that do not result from altering the DNA sequence [12]. The epigenome plays a crucial role in DNA-based biological processes, such as replication, transcription, and DNA repair. Consequently, aberrant epigenetic alterations are associated with the development and progression of numerous diseases. Mutations of epigenetic regulators are prevalent in cancer, and abnormal epigenetic changes have been acknowledged as key drivers of tumorigenesis [13]. Therefore, understanding epigenetic processes may provide effective treatment strategies for different types of diseases. Among numerous posttranslational modification epigenetic modulators, histone protein acetylation is catalyzed by histone acetyltransferases (HATs) and histone deacetylases (HDACs), which function as “writers” and “erasers” that mediate the addition or removal of acetyl groups from the lysine residues of histone proteins [14]. Although HDACs were primarily identified to deacetylate histones, the following discoveries have revealed the non-histone substrates of HDACs, such as transcription factors, heat shock proteins, and structural proteins [15].

HDAC8 is the latest member of class I HDAC. Various studies have demonstrated the pathological role of HDAC8 in diseases, including cancer. The aberrant overexpression of HDAC8 has been observed in various cancers, such as gastric cancer [16], hepatocellular carcinoma (HCC) [17], oral squamous cell carcinoma (OSCC) [18], and childhood acute lymphoblastic leukemia (ALL) [19]. In particular, HDAC8 expression is correlated with poor outcomes in neuroblastoma [20] and breast cancer [21]. Like many of the other HDACs, HDAC8 plays a crucial role in various aspects of cancer development by interacting with histone and non-histone proteins. It has been found to be involved in sustaining cell proliferation, suppressing apoptosis, activating invasion and metastasis, evading the immune response, and developing drug resistance, [22]. Targeting HDAC8 is an attractive target for cancer therapy, and a myriad number of inhibitors have been developed thus far [23]. PCI-34051 is one of the most widely used HDAC8-selective inhibitors (HDAC8is), with 200-fold selectivity over other class I HDACs, and it has shown significant anti-cancer effects in various cancer cells [24,25].

Because HIF-1α is upregulated in melanoma and contributes to the malignant transformation of melanocytes [26], we sought to identify a novel epigenetic modulator of HIF-1α, thereby providing a therapeutic target in melanoma. Here, we evaluated the effects of several HDACis and found that PCI-34051 most effectively suppresses HIF-1α expression in melanoma. A further investigation validated the regulatory role of HDAC8 in enhancing HIF-1α expression. HDAC8 deacetylates HIF-1α, thereby increasing protein stability and promoting transcriptional activity. Moreover, we verified that HDAC8 promotes the progression of melanoma by enhancing cell proliferation and metastasis. Lastly, we corroborated our results with samples from patients with melanoma, which showed that the upregulation of HDAC8 and HIF-1α is correlated with poor prognosis in melanoma. Collectively, these findings reveal HDAC8 as a novel epigenetic modulator of HIF-1α and present a novel therapeutic strategy in treating patients with melanoma.

## 2. Materials and Methods

### 2.1. Reagents

HDAC inhibitors (Table 1) were solubilized in dimethyl sulfoxide (DMSO) (Sigma Chemical, St Louis, MO, USA). The primary antibodies used include the following: α-tubulin (sc-32293, Santa Cruz Biotechnology, Santa Cruz, CA, USA), histone H3 (sc-10809, Santa Cruz), GAPDH (AP0066, Bioworld Technology, Bloomington, MN, USA), SMC3 (A300-060A-M, Bethyl Laboratories, Montgomery, TX, USA), ac-SMC3 (MABE1073, Millipore, Burlington, MA, USA), ac-histone H3 (06-599, Millipore), HDAC8 (ab187139, Abcam, Cambridge, UK), HIF-1α (610959, BD PharMingen, San Diego, CA, USA), GLUT1 (#12939, Cell Signaling Technology, Danvers, MA, USA), and HK2 (#2867, Cell Signaling Technology).

### 2.2. Cell Line and Culture

The human melanoma cell line and A2058, SK-MEL-2, and SK-MEL-28 were purchased from the American Type Culture Collection (ATCC). The melanoma cells were cultured in Gibco Minimum Essential Media (WelGene, Daegu, Republic of Korea). HEK293T cells were purchased from the American Type Culture Collection (ATCC) and maintained in Dulbecco’s modified Eagle’s medium (Sigma-Aldrich, St. Louis, MO, USA). Both media were supplemented with 10% fetal bovine serum, 100 U/mL penicillin, and 100 µg/mL streptomycin. The cell lines were maintained in 5% CO_2_ at 37 °C in humidified incubators and subcultured every 3–4 days. Hypoxia was induced using cobalt chloride (CoCl_2_) (Sigma-Aldrich, St. Louis, MO, USA) or by placing the cells in a hypoxia induction chamber (#27310, STEMCELL Technologies Inc., Vancouver, BC, Canada) loaded with mixed gas containing 1% O_2_, 5% CO_2_, and 94% N_2_.

### 2.3. CRISPR/Cas9-Mediated Knockout (KO) of HDAC8 Gene

HDAC8 KO stable cells were generated using gRNAs targeting HDAC8. The gRNAs were cloned into the LCV2 lentiviral vector. The lentivirus was made with HEK293T cells. On a 6-well plate, 5 × 10^5^ HEK293T cells were seeded and transfected with 1.4 µg of the gRNA lentiviral vector, 1.2 µg of the packaging vector psPAX2, and 0.4 µg of the envelope vector pMD2.G. To mediate the transfection, 7.5 µL of polyethyleneimine (Polysciences, Inc., Warrington, PA, USA) was added. Then, 24 h after the transfection, the supernatant was collected and transduced into A2058 cells with 4 µg/mL of polybrene (Sigma-Aldrich, St. Louis, MO, USA). The KO cells were selected by treating the cells with 2 µg/mL of puromycin (Enzo Life Sciences, Inc., Farmingdale, NY, USA). To generate the HDAC8 rescue cell lines, the A2058 cells were first transfected with gHDAC8 to generate HDAC8 KO cell lines. Following puromycin selection, the HDAC8 KO cells were transfected with either wild-type (WT) HDAC8 or catalytically dead Y306F-mutant (MT) HDAC8. The gRNAs used are listed in Table 2. All the experiments were approved by the Institutional Biosafety Committee of Yonsei University (IBC-A-202212-326-01).

### 2.4. Cell Growth and Viability Assay

Cell growth and viability were determined using a CCK-8 assay (Dojindo Molecular Technologies, Inc., Kumamoto, Japan). SK-MEL-2, SK-MEL-28, and A2058 cells were seeded at 3 × 10^3^ cells per well in 96-well plates containing 130 μL medium. After overnight incubation, the cells were treated with 0.1% DMSO or PCI-34051 at various concentrations (0.1, 0.3, 1, 3, 10, 30, 100 µM) for 24 h, 48 h, and 72 h. The A2058 HDAC8 KO and overexpression (OE) cells were seeded at 3 × 10^3^ cells per well in 96-well plates containing 130 μL medium and incubated for 24 h, 48 h, and 72 h. Hypoxia was induced using 100 µM CoCl_2_ or by placing the cells in a hypoxia induction chamber. Then, 13 μL of the CCK-8 reagent was added to each well for 3 h. Absorbance was measured at 450 nm using a multimode microplate reader (Tecan Group, Ltd., Mannedorf, Switzerland). The results were quantified relative to the control, and they are presented as percentages. The half-maximal cell viability inhibition concentration (IC_50_) and half-maximal growth inhibition concentration (GI_50_) were determined using GraphPad Prism ver. 7.0 software (GraphPad Software, San Diego, CA, USA).

### 2.5. Colony Formation Assay

A2058 cells were seeded at 1 × 10^3^ cells per well in a six-well plate. After overnight incubation, the cells were treated with 0.1% DMSO or 20 µM PCI-34051. The A2058 HDAC8 KO and OE cells were seeded at 1 × 10^3^ cells per well in a six-well plate. Hypoxia was induced using 20 µM CoCl_2_ or by placing the cells in a hypoxia induction chamber. The cells were incubated for another 12 days, and colonies were stained with 1 mL of 0.05% crystal violet solution (Sigma-Aldrich, St. Louis, MO, USA) for 5 min.

### 2.6. Luciferase Reporter Assay

A2058 cells were seeded at 3 × 10^5^ cells per well in a six-well plate. After overnight incubation, the cells were transfected with hypoxic response element (HRE)-Luc reporter gene with polyethyleneimine. Hypoxia was induced using 100 µM CoCl_2_. After 24 h, luciferase activities were measured using the Dual Luciferase Assay System (Promega Corporation, Madison, WI, USA) and a multimode microplate reader.

### 2.7. Wound Healing Assay

A2058 cells were seeded at 1 × 10^6^ cells per plate in a six-well plate. After overnight incubation, a linear scratch was made using a 200 μL pipette tip. Cell debris was removed by washing the cells with a serum-free medium. Then, the cells were treated with 0.1% DMSO or 20 µM PCI-34051 for 24 h. Hypoxia was induced using 100 µM CoCl_2_. A2058 HDAC8 KO and OE cells were seeded at 1 × 10^6^ cells per well in a six-well plate. After overnight incubation, a linear wound midline was made using a 200 μL pipette tip. Hypoxia was induced using 100 µM CoCl_2_. Cell debris was removed by washing it with a serum-free medium. An inverted microscope (Carl Zeiss AG, Feldbach, Switzerland) was used to observe the wound closure, and ImageJ software (National Institutes of Health, Bethesda, MD, USA) was used for quantification.

### 2.8. Transwell Migration Assay

A2058 cells were seeded at 3 × 10^4^ cells per well in a 400 μL serum-free medium to the upper chamber of a Transwell insert plate (PET membrane, 8 μM pore, SPL 36224, Republic of Korea). Then, 500 μL of a medium containing 10% FBS was added to the lower chamber. After overnight incubation, 20 μM PCI-34051 was applied to the lower well. A2058 HDAC8 KO and OE cells were seeded at 3 × 10^4^ cells per well in a 400 μL serum-free medium to the upper chamber, and 500 μL of medium containing 10% FBS was added to the lower chamber of the Transwell insert plate. Hypoxia was induced using 100 µM CoCl_2_. After 48 h incubation, the migrated cells were fixed and stained using 0.1% crystal violet solution containing 20% methanol. To visualize the stained cells, a light microscope with iSolution Lite (IMT i-Solution Inc., Vancouver, BC, Canada) was used. For quantification, the stained cells were solubilized with 10% acetic acid. Absorbance was measured at 560 nm using a multimode microplate reader.

### 2.9. Western Blot Analysis

A2058 cells were seeded at 5 × 10^5^ cells per well in a six-well plate. After overnight incubation, the cells were treated with 0.1% DMSO or 20 µM PCI-34051 for 24 h. A2058 HDAC8 KO and OE cells were seeded at 5 × 10^5^ cells per well in a six-well plate and incubated for 24 h. Hypoxia was induced using 100 µM CoCl_2_ for 12 h or by placing the cells in a hypoxia induction chamber. The cells were washed with ice-cold 1× PBS and lysed with 100 µL lysis buffer (50 mM Tris-HCl (pH 7.4), 150 mM NaCl, 25 mM glycerol phosphate, 25 mM NaF, 5 mM EGTA, 1 mM EDTA, 1 mM DTT, 1% NP-40, 1 µg/mL leupeptin, 1 µg/mL aprotinin, 1 mM PMSF, 1 mM benzamidine). The protein concentrations of the cell lysates were measured using a Bradford assay. Samples were prepared with a 5× sample buffer and boiled for 5 min. The prepared samples were loaded onto a polyacrylamide gel and subjected to SDS-PAGE. Then, the gel was transferred onto a nitrocellulose membrane. After blocking the membrane with 5% skim milk, it was incubated with primary antibodies at 4 °C overnight. Membranes were washed with 0.1% Tween-20/PBS and incubated with an anti-mouse or anti-rabbit secondary antibody coupled to HRP at room temperature for 3 h. The ECL Western blotting analysis system (Thermo Scientific Pierce, Waltham, MA, USA) was used for detection.

### 2.10. Co-Immunoprecipitation Assay (Co-IP)

A2058 cells were seeded at 1 × 10^6^ cells per plate in a cell culture dish. After overnight incubation, the A2058 cells were treated with 100 µM CoCl_2_ for 12 h to induce hypoxia. HEK293T cells were seeded at 1 × 10^6^ cells per plate in a cell culture dish. After overnight incubation, the HEK293T cells were transfected with 1 µg of FLAG-HDAC8 and 1 µg of HA-HIF-1α. Polyethylenimine was added to mediate the transfection. The cells were washed with ice-cold 1× PBS and lysed with 100 µL lysis buffer. Then, 1000 µg of cell lysates was incubated with primary antibodies and 20 µL of A/G beads (Santa Cruz Biotechnology, Santa Cruz, CA, USA) at 4 °C overnight. The lysate mixtures were washed with lysis buffer (300 mM NaCl), prepared with 40 µL of 5× sample buffer, and boiled for 5 min to be subjected to SDS-PAGE.

### 2.11. RNA Extraction and qRT-PCR

A2058 cells were seeded at 1 × 10^6^ cells per plate in a 60 mm cell culture plate. After overnight incubation, the cells were treated with 0.1% DMSO or 20 µM PCI-34051 for 24 h. A2058 HDAC8 KO and OE cells were seeded at 1 × 10^6^ cells per plate in a 60 mm cell culture plate. Hypoxia was induced using 100 µM CoCl_2_ for 6 h or by placing the cells in a hypoxia induction chamber. The total RNA from the cells was extracted using a FavorPrep^TM^ Blood/Cultured Cell Total RNA Mini Kit (Favorgen Biotech Corporation, Ping-Tung, Taiwan). One microgram of total RNA was reverse-transcribed into cDNA using an EasyScript™ cDNA synthesis kit (TransGen Biotech, Beijing, China). One microliter of cDNA was amplified using 10 µL of TOPreal™ qPCR 2X PreMIX SYBR green reagent (Enzynomics, Daejeon, Republic of Korea). A qPCR analysis was performed using the Applied Biosystems 7500 System (Applied Biosystems, Foster City, CA, USA). The mRNA values were normalized by GAPDH expression levels. The primers used are listed in Table 3.

### 2.12. Analysis of Online Databases

The association between patient survival and the expressions of *HDAC8* and *HIF1A* was analyzed using gene expression profiles from the NCBI Gene Expression Omnibus (GEO) database (GSE65904, *n* = 214 [27]) using the online website http://bioinfo.henu.edu.cn/ accessed on 1 November 2022. The expressions of *HDAC8* and *HIF1A* based on sample types and the gene expression correlation between *HDAC8* and *HIF1A* were analyzed using gene expression profiles from The Cancer Genome Atlas (TCGA) database (*n* = 473) using the website http://ualcan.path.uab.edu/index.html accessed on 1 November 2022 [28].

### 2.13. Statistical Analysis Figures, Tables, and Schemes

All data are expressed as means ± standard deviation (SD) of more than three independent experiments. Statistical significance was determined using an unpaired two-tailed Student’s t-test or a one-way analysis of variance (ANOVA) with a post hoc analysis under Turkey’s multiple comparison test using GraphPad Prism software 7.0 (GraphPad Software, San Diego, CA, USA). Differences were considered statistically significant at *p*-values < 0.05.

## 3. Results

### 3.1. HDAC8 Upregulates HIF-1α Expression in Melanoma

**Figure 1:**
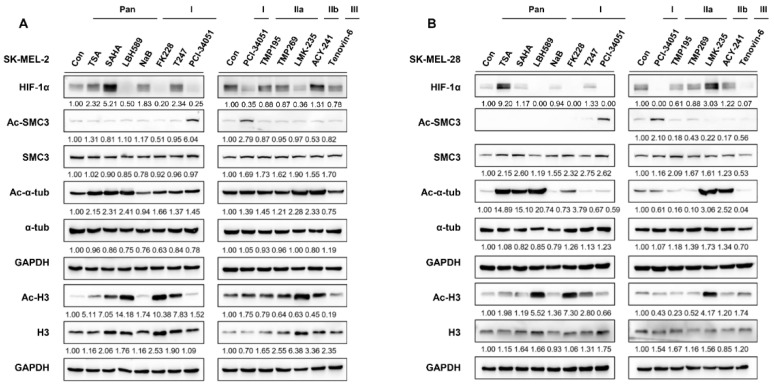
HDAC inhibitors differentially regulate HIF-1α expression. (**A**,**B**) HIF-1α expression upon HDAC inhibitor treatment. Melanoma cells (**A**) SK-MEL-2 and (**B**) SK-MEL-28 were cultured with 0.1% DMSO (control; con) or respective HDAC inhibitors. Acetyl-SMC3 (Ac-SMC3) levels were semi-quantified relative to SMC3 protein levels. Acetyl-α-tubulin (Ac-α-tub) levels were semi-quantified relative to α−tubulin (α−tub). Acetyl-histone 3 (Ac-H3) levels were semi-quantified relative to histone 3 (H3) levels. HIF-1α, SMC3, α−tub, and H3 protein expression levels were semi-quantified relative to the loading control, GAPDH. The uncropped Western Blot images can be found in Figure S3.

**Figure 2:**
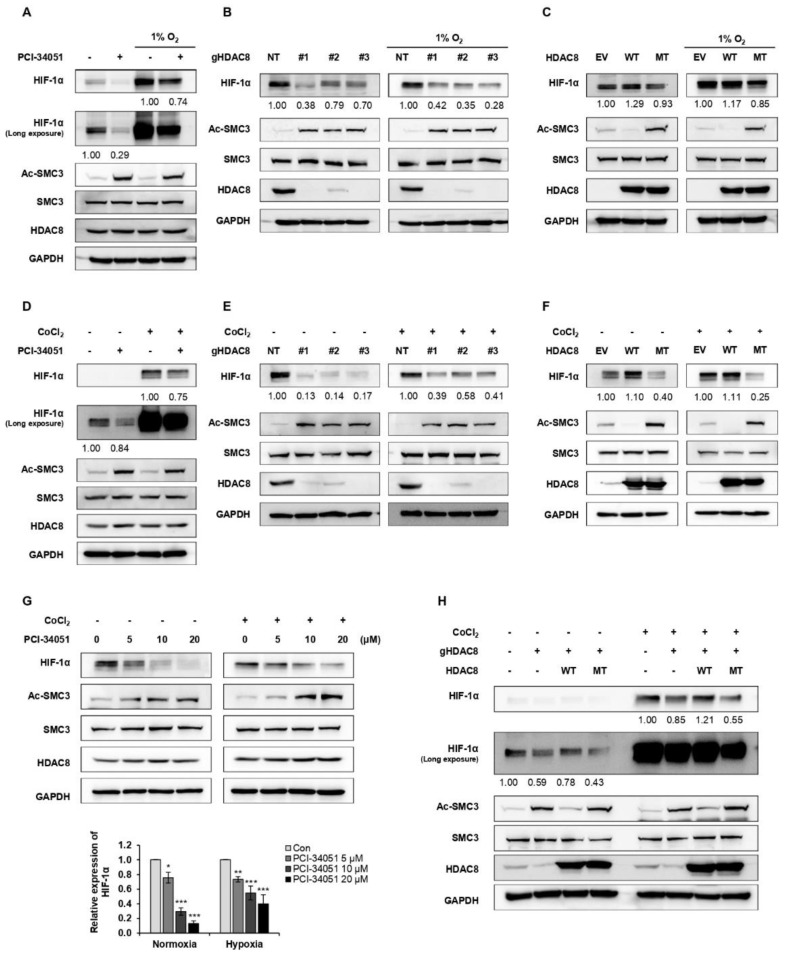
HDAC8 upregulates HIF-1α expression. (**A**–**C**) Alteration in HIF-1α upon HDAC8 activity inhibition, depletion, or overexpression under normoxia and hypoxia induced by incubating cells in 1% O_2_. (**A**) Immunoblotting of HIF-1α upon PCI-34051 treatment in A2058 cells. A2058 cells were treated with 0.1% DMSO (control; con) or 20 μM PCI-34051 for 24 h under normoxic and hypoxic conditions. (**B**) Immunoblotting of HIF-1α in non-target (NT) control and three gHDAC8 (HDAC8 KO) A2058 cells under normoxic and hypoxic conditions. (**C**) Immunoblotting of HIF-1α in empty vector (EV) control and A2058 cells stably overexpressing wild-type (WT) HDAC8 or catalytically dead Y3060F-mutant (MT) HDAC8 under normoxic and hypoxic conditions. (**D**–**F**) Alteration in HIF-1α upon HDAC8 activity inhibition, depletion, or overexpression under normoxia and hypoxia induced by treating the cells with 100 μM CoCl_2_ for 12 h. (**D**) Immunoblotting of HIF-1α upon PCI-34051 treatment in A2058 cells. A2058 cells were treated with 0.1% DMSO or 20 μM PCI-34051 for 24 h under normoxic and hypoxic conditions. (**E**) Immunoblotting of HIF-1α in NT control and three gHDAC8 (HDAC8 KO) A2058 cells under normoxic and hypoxic conditions. (**F**) Immunoblotting of HIF-1α in EV control and A2058 cells stably overexpressing WT HDAC8 or MT HDAC8 under normoxic and hypoxic conditions. (**G**) Immunoblotting of HIF-1α upon increasing doses of PCI-34051 treatment. A2058 cells were treated with 0.1% DMSO or PCI-34051 at respective concentrations (0, 5, 10, 20 μM) for 24 h under normoxic and hypoxic conditions. Hypoxia was induced by treating the cells with 100 μM CoCl_2_ for 12 h. (**H**) Immunoblotting of HIF-1α in HDAC8-rescued A2058 cells. HDAC8 KO A2058 cells were transfected with WT HDAC8 or MT HDAC8 vector and cultured under normoxic and hypoxic conditions. Hypoxia was induced by treating the cells with 100 μM CoCl_2_ for 12 h. Protein expression levels were semi-quantified relative to the loading control, GAPDH. Data are presented as the mean ± SD of three independent experiments. * *p* < 0.05, ** *p* < 0.01, or *** *p* < 0.001 vs. control determined by one-way ANOVA. The uncropped Western Blot images can be found in Figure S4.

To determine which HDAC effectively regulates HIF-1α expression, we treated melanoma cell lines, SKMEL-2 and SKMEL-28, with different HDAC inhibitors (Table 1) and examined the changes in HIF-1α expression levels. Among the inhibitors tested, LBH589 (panobinostat), FK228 (romidepsin), and PCI-34051 efficiently suppressed the protein level of HIF-1α (Figure 1A,B). Studies have demonstrated that HDAC1 and HDAC2 enhance the protein stability of HIF-1α [29,30]. Consistent with previous findings, FK228, a potent HDAC1 and HDAC2 inhibitor, significantly reduced the HIF-1α protein levels in melanoma. Moreover, HIF-1α levels were downregulated when treated with a pan-HDAC inhibitor, LBH589. Surprisingly, PCI-34051, a selective inhibitor of HDAC8, greatly reduced HIF-1α expression regardless of the normoxic or hypoxic conditions, suggesting that it is a novel epigenetic modulator of HIF-1α (Figure 2A). We further verified the regulatory role of HDAC8 by examining the HIF-1α expression levels in an HDAC8 knockout (KO) and overexpression (OE) A2058 cell line under normoxia and hypoxia induced by depleting O_2_ levels (Figure 2B,C). HDAC8 KO cells were established by targeting HDAC8 (gHDAC8) using the CRISPR-Cas9 system. Similar to the PCI-34051 results, the genetic ablation of HDAC8 greatly reduced HIF-1α levels under both normoxic and hypoxic conditions in melanoma cells (Figure 2B). Furthermore, the overexpression of wild-type (WT) HDAC8 increased HIF-1α levels, while the catalytically dead Y3060F-mutant (MT) HDAC8 [31] suppressed its expression (Figure 2C). Similar patterns of HIF-1α were also shown under cobalt chloride (CoCl_2_)-induced hypoxic conditions (Figure 2D–F). Next, we treated the A2058 cells with increasing doses of PCI-34051. The HIF-1α levels decreased in a dose-dependent manner in both normoxic and CoCl_2_-induced hypoxic conditions (Figure 2G). Lastly, re-introducing wild-type HDAC8 into the HDAC8 KO A2058 cells rescued HIF-1α levels, while mutant HDAC8 was unable to restore HIF-1α levels (Figure 2H). Together, these results show that HDAC8 positively regulates HIF-1α expression.

### 3.2. HDAC8 Deacetylates HIF-1α and Enhances Its Protein Stability

**Figure 3:**
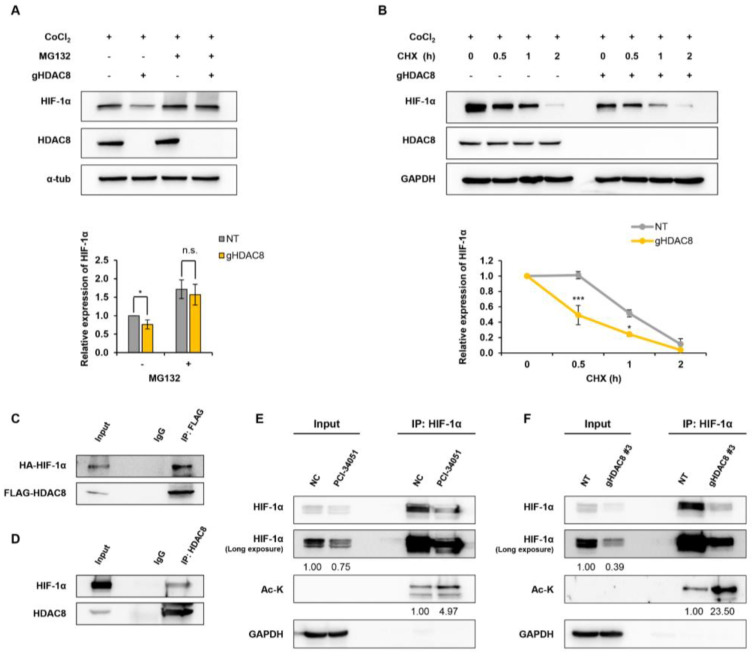
HDAC8 deacetylates HIF-1α and enhances HIF-1α protein stability. (**A**,**B**) Immunoblot of changes in HIF-1α protein stability in A2058 cells treated with 100 μM CoCl_2_ for 12 h (h) to mimic hypoxic conditions. (**A**) Immunoblotting of HIF-1α treated with MG132. Non-target (NT) control and gHDAC8 (HDAC8 KO) A2058 cells were treated with 5 μM MG132 for 1 h under CoCl_2_-induced hypoxic conditions. (**B**) Immunoblotting of HIF-1α treated with cycloheximide (CHX). NT and HDAC8 KO A2058 cells were treated with 5 μM CHX for the indicated time (0, 0.5, 1, 2 h) under CoCl_2_-induced hypoxic conditions. (**C**) Co-immunoprecipitation (Co-IP) assay of FLAG-tagged HDAC8 (FLAG-HDAC8) and HA-tagged HIF-1α (HA-HIF-1α). HEK-293T cells were transfected with FLAG-HDAC8 and HA-HIF-1α vectors. Immunoprecipitation (IP) assays were performed with IgG or anti-FLAG antibodies, followed by immunoblotting with antibodies against FLAG and HA. (**D**) Co-IP assay of endogenous HDAC8 and HIF-1α. A2058 cells were cultured in hypoxic conditions induced by treating the cells with 100 μM CoCl_2_ for 12 h. IP assays were performed with IgG or HDAC8, followed by immunoblotting with antibodies against HDAC8 and HIF-1α. (**E**,**F**) Immunoblotting of immunoprecipitated HIF-1α lysine acetylation levels in hypoxic conditions induced by treating the cells with 100 μM CoCl_2_ for 12 h. (**E**) Elevated HIF-1α lysine acetylation upon PCI-34051 treatment. A2058 cells were treated with 0.1% DMSO (negative control; NC) or 20 μM PCI-34051 for 24 h under hypoxic conditions. IP assays were performed with IgG or anti-HIF-1α antibodies, followed by immunoblotting with antibodies against HIF-1α and acetyl-lysine (Ac-K). (**F**) Elevated HIF-1α lysine acetylation upon HDAC8 knockout. NT and HDAC8 KO A2058 cells were cultured under hypoxic conditions. IP assays were performed with IgG or anti-HIF-1α antibodies followed by immunoblotting with antibodies against HIF-1α and Ac-K. Protein expression levels were semi-quantified relative to the loading control, GAPDH or α-tubulin. Data are presented as the mean ± SD of three independent experiments. * *p* < 0.05, or *** *p* < 0.001 vs. control determined by one-way ANOVA. The uncropped Western Blot images can be found in Figure S5.

HDAC8 modulates the expressions of target genes by regulating either its transcriptional level or posttranscriptional level [22]. To test whether HDAC8 regulates HIF-1α at the posttranscriptional level, A2058 cells were treated with a 26S proteasome-specific inhibitor, MG132, and HIF-1α protein levels were observed. The results show that the HIF-1α protein levels suppressed upon HDAC8 depletion were restored in the presence of MG132, strongly suggesting that HDAC8 regulated the protein stability of HIF-1α (Figure 3A). For further verification, a cycloheximide (CHX) pulse-chase experiment was performed. The degradation rate of HIF-1α was calculated by treating A2058 cells with CHX and harvesting cell lysates 0.5, 1, and 2 h after treatment. Compared to the control cells, HIF-1α proteins degraded faster in the HDAC8 KO A2058 cells, further confirming that HDAC8 regulated HIF-1α protein stability (Figure 3B).

Previous reports have shown that HDACs interact with HIF-1α and regulate the protein stability of HIF-1α [32]. Thus, we first evaluated the protein–protein interaction between HDAC8 and HIF-1α. HEK-293T cells were co-transfected with FLAG-HDAC8 and HA-HIF-1α plasmids. A co-immunoprecipitation assay revealed that HDAC8 interacted with HIF-1α (Figure 3C). We further confirmed endogenous HDAC8 and HIF-1α interaction in the A2058 cells (Figure 3D). Given that HIF-1α interacted with HDAC8, we then assessed whether HDAC8 mediates the deacetylation of HIF-1α. The genetic ablation or pharmacological inhibition of HDAC8 greatly increased the levels of acetylated HIF-1α compared to those of the control cells (Figure 3E,F). Collectively, these results demonstrate that HDAC8 deacetylates HIF-1α and enhances its protein stability, thereby stimulating HIF-1α accumulation.

### 3.3. HDAC8 Regulates HIF-1α Transcriptional Activity

**Figure 4:**
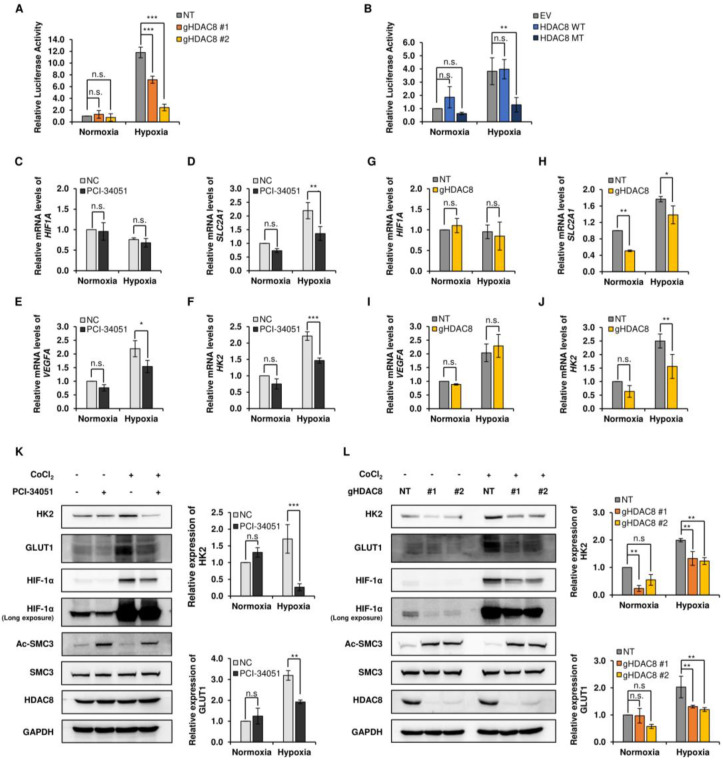
Transcriptional activity of HIF-1α is decreased in the suppression of HDAC8. (**A**,**B**) Luciferase reporter assays in A2058 cells. (**A**) Non-target (NT) control and two gHDAC8 (HDAC8 KO) A2058 cells were transfected with the hypoxic response element (HRE)-Luc reporter gene under normoxic and hypoxic conditions and assayed for luciferase activity. Hypoxia was induced by treating the cells with 100 μM CoCl_2_ for 12 h (h). (**B**) Empty vector (EV) control and A2058 cells stably overexpressing WT HDAC8 or MT HDAC8 were transfected with HRE-Luc reporter gene under normoxic and hypoxic conditions and assayed for luciferase activity. Hypoxia was induced by treating the cells with 100 μM CoCl_2_ for 6 h. (**C**–**F**) qRT-PCR analysis of HIF-1α downstream target genes in A2058 cells treated with 0.1% DMSO or 20 μM PCI-34051 for 24 h. Hypoxia was induced by treating the cells with 100 μM CoCl_2_ for 6 h. mRNA levels of respective target genes were evaluated via qRT-PCR and semi-quantified relative to GAPDH gene expression. The relative mRNA levels of (**C**) *HIF1A*, (**D**) *SLC2A1*, (**E**) *VEGFA*, and (**F**) *HK2* were compared to negative control (NC) upon PCI-34051 treatment under normoxia and hypoxia. (**G**–**J**) qRT-PCR analysis of HIF-1α downstream target genes in NT and HDAC8 KO A2058 cells. Hypoxia was induced by treating the cells with 100 μM CoCl_2_ for 6 h. mRNA levels of respective target genes were evaluated via qRT-PCR and semi-quantified relative to GAPDH gene expression. The relative mRNA levels of (**G**) *HIF1A*, (**H**) *SLC2A1*, (**I**) *VEGFA*, and (**J**) *HK2* were compared to NT upon HDAC8 KO under normoxia and hypoxia. (**K**,**L**) Immunoblotting of HIF-1α downstream target genes under normoxic and hypoxic conditions. Hypoxia was induced by treating the cells with 100 μM CoCl_2_ for 12 h. (**K**) Immunoblotting of HIF-1α downstream target genes upon PCI-34051 treatment. A2058 cells were treated with 0.1% DMSO or 20 μM PCI-34051 for 24 h under normoxic and CoCl_2_-induced hypoxic conditions. (**L**) Immunoblotting of HIF-1α downstream target genes in NT and two HDAC8 KO A2058 cells under normoxic and CoCl_2_-induced hypoxic conditions. Protein expression levels were semi-quantified relative to the loading control, GAPDH. Data are presented as the mean ± SD of three independent experiments. * *p* < 0.05, ** *p* < 0.01, or *** *p* < 0.001 vs. control determined by one-way ANOVA. n.s., nonsignificant. The uncropped Western Blot images can be found in Figure S6.

HIF-1α is a transcription factor that regulates numerous cellular pathways involved in tumor progression, such as glycolytic metabolism, metastasis, and angiogenesis [33]. Next, to assess whether HDAC8 also regulates the transcriptional activity of HIF-1α, we performed a luciferase activity assay in HDAC8 KO and OE cell lines. A2058 cells were transfected with hypoxic response element (HRE)-Luc, and luciferase activity was measured under normoxic and CoCl_2_-induced hypoxic conditions. HDAC8 depletion strongly suppressed HRE-driven luciferase activity in hypoxia (Figure 4A,B). Furthermore, MT HDAC8 overexpression significantly suppressed luciferase activity under hypoxic conditions, whereas WT HDAC8 showed no significant differences. Next, we examined the mRNA levels of HIF-1α and its target genes upon HDAC8 suppression. While CoCl_2_-induced hypoxia upregulated the mRNA levels of HIF-1α target genes, solute carrier family 2 member 1 (*SLC2A1*), vascular endothelial growth factor A (*VEGFA*), and hexokinase 2 (*HK2*), PCI-34051 treatment significantly inhibited this effect (Figure 4C–F and Supplementary Figure S1A–D). Similarly, in the HDAC8 KO cells, the mRNA levels of *SLC2A1* and *HK2* were downregulated upon the genetic ablation of HDAC8 (Figure 4G–J). The following results were further confirmed through a Western blot analysis, which showed reduced protein levels of HK2 and glucose transporter 1 (GLUT1, encoded by *SLC2A1*) in the PCI-34051 treated and HDAC8 KO cells (Figure 4K,L). Surprisingly, the mRNA level of *HIF1A* did not show significant changes upon HDAC8 inhibition or HDAC8 depletion (Figure 4C,G). Therefore, these data suggest that HDAC8 posttranscriptionally stabilizes HIF-1α by catalyzing its deacetylation. In turn, the stabilized HIF-1α shows enhanced transcriptional activity on its target genes.

### 3.4. HDAC8 Promotes Cell Proliferation and Enhances Migration in Melanoma

**Figure 5:**
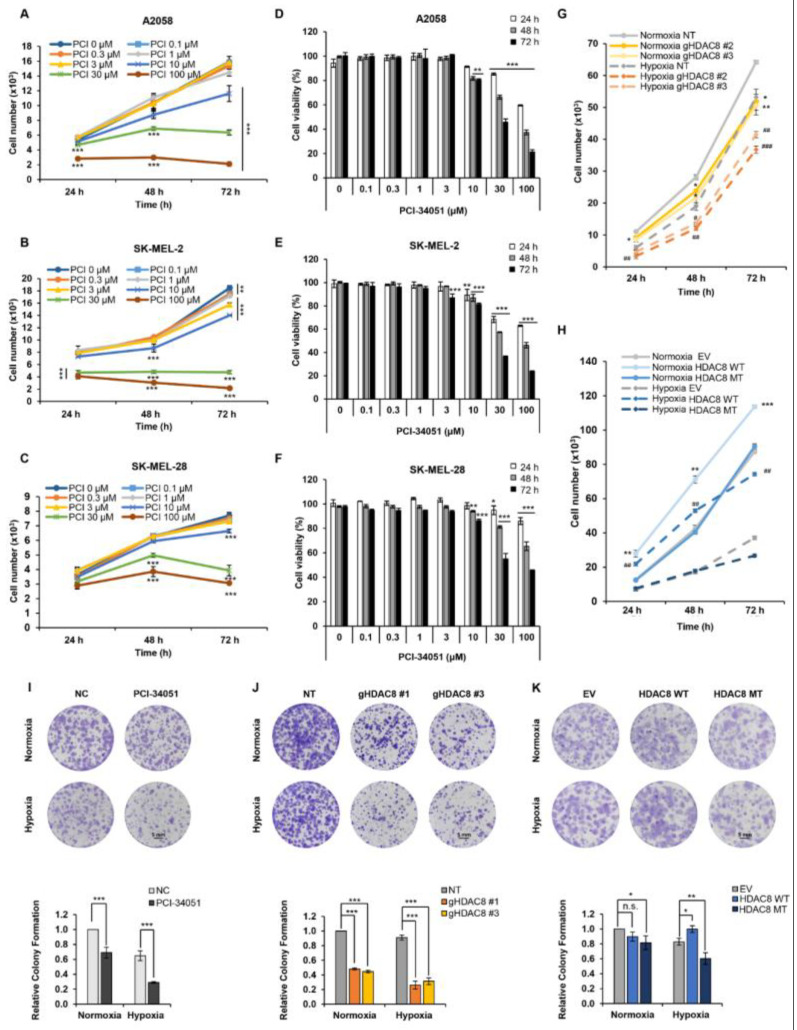
HDAC8 regulates cell proliferation in melanoma cells. Cell growth curves of (**A**) A2058, (**B**) SK-MEL-2, and (**C**) SK-MEL-28 melanoma cells treated with 0.1% DMSO (PCI 0 μM) or described concentrations of PCI-34051 for 24 h (h), 48 h, and 72 h under normoxic conditions. Relative cell viability of (**D**) A2058, (**E**) SK-MEL-2, and (**F**) SK-MEL-28 melanoma cells with 0.1% DMSO or described concentrations of PCI-34051 for 24 h, 48 h, and 72 h under normoxic conditions. (**G**) Growth curves of non-target (NT) control and gHDAC8 (HDAC8 KO) A2058 cells cultured for 24 h, 48 h, and 72 h under normoxic and hypoxic conditions. (**H**) Growth curves of empty vector (EV) control and A2058 cells stably overexpressing WT HDAC8 or MT HDAC8 cultured for 24 h, 48 h, and 72 h under normoxic and hypoxic conditions. Cell growth and viability were measured using a CCK-8 assay. Hypoxia was induced by treating the cells with 100 μM CoCl_2_. Data are presented as the mean ± SD of three independent experiments. * *p* < 0.05, ** *p* < 0.01, or *** *p* < 0.001 vs. normoxic control determined by Student’s *t*-test. ^#^ *p* < 0.05, ^##^ *p* < 0.01 or ^###^ *p* < 0.001 vs. hypoxic control determined by Student’s *t*-test. Colony formation of (**I**) A2058 cells treated with 0.1% DMSO (negative control; NC) or 5 μM PCI-34051, (**J**) NT control and HDAC8 KO A2058 cells, and (**K**) EV control and A2058 cells stably overexpressing WT HDAC8 or MT HDAC8 incubated for 12 days under normoxic and hypoxic conditions. Scale bar = 5 mm. Hypoxia was induced by treating the cells with 20 μM CoCl_2_. Data are presented as the mean ± SD of three independent experiments. * *p* < 0.05, ** *p* < 0.01, or *** *p* < 0.001 vs. control determined by one-way ANOVA. n.s., nonsignificant.

Here, we revealed that HDAC8 deacetylates HIF-1α, thereby enhancing its transcriptional activity involved in various oncogenic signaling [34]. Although studies have elucidated the crucial role of HDAC8 in promoting tumor progression [22], in melanoma, the tumorigenic role of HDAC8 remains controversial. In fact, the expression of HDAC8 has been reported to be associated with improved survival in patients with melanoma [35]. Our results, which point to another tumorigenic role of HDAC8 in melanoma, prompted our investigation of HDAC8 in melanoma progression. First, we evaluated whether the pharmacological inhibition of HDAC8 would negatively affect the proliferation of melanoma cells (Figure 5A–F). A CCK-8 assay was performed to estimate the cell growth and viability of melanoma cell lines. PCI-34051 greatly repressed cell growth and viability with GI_50_ values of 15.38 µM, 14.39 µM, and 17.62 µM and IC_50_ values of 21.03 µM, 15.72 µM, and 21.64 µM in A2058, SK-MEL-2, and SK-MEL-28, respectively (Table 4). Next, we observed the effect of HDAC8 KO or OE on the proliferation of A2058 cells. The CCK-8 results demonstrated that HDAC8 KO suppressed cell proliferation, while WT HDAC8 enhanced cell proliferation under both normoxic and hypoxic conditions (Figure 5G,H and Supplementary Figure S2A). Then, a colony formation assay was performed to assess the long-term effects of HDAC8 on cell proliferation. PCI-34051 and HDAC8 KO significantly suppressed the ability of A2058 cells to form colonies under both normoxic and hypoxic conditions (Figure 5I–K and Supplementary Figure S2B). While the effect of WT HDAC8 and MT HDAC8 was minimal in normoxia, WT HDAC8 significantly enhanced the colony formation capacity under CoCl_2_-induced hypoxic conditions. Collectively, these results indicate that HDAC8 upregulates both the short- and long-term proliferation of melanoma cells.

**Figure 6:**
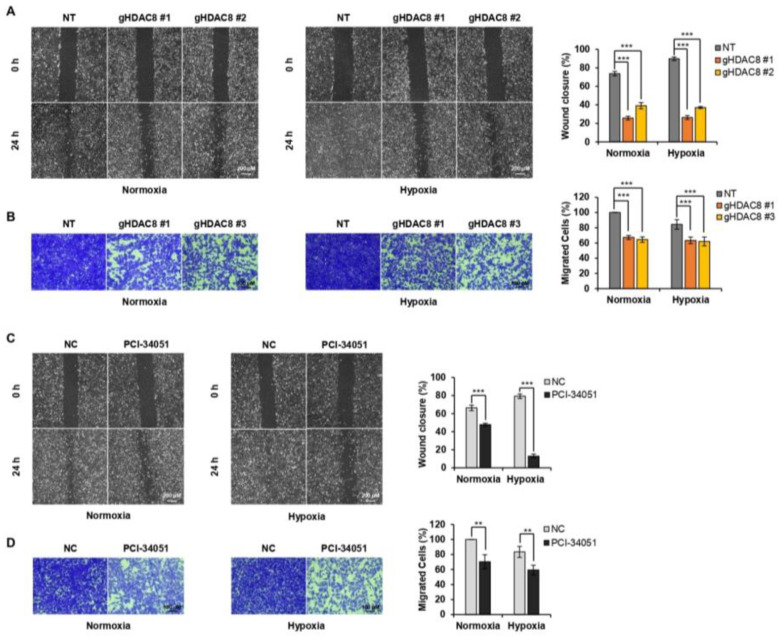
HDAC8 depletion or inhibition suppresses metastasis in melanoma cells. (**A**) Wound-healing assay of non-target (NT) and gHDAC8 (HDAC8 KO) A2058 cells, scratched and incubated for 24 h (h) under normoxic and hypoxic conditions. The width of the scratch was calculated and photographed. Scale bar = 200 μm. (**B**) Transwell assay of NT and HDAC8 KO A2058 cells incubated for 48 h under normoxic and hypoxic conditions. Migrated cells were stained and photographed. Scale bar = 100 μm. (**C**) Wound-healing assay of A2058 cells, scratched and treated with 0.1% DMSO (negative control; NC) or 20 μM PCI-34051 for 24 h under normoxic and hypoxic conditions. The width of the scratch was calculated and photographed. Scale bar = 200 μm. (**D**) Transwell assay of A2058 cells treated with 0.1% DMSO or 20 μM PCI-34051 for 48 h under normoxic and hypoxic conditions. Migrated cells were stained and photographed. Scale bar = 100 μm. Hypoxia was induced by treating the cells with 100 μM CoCl_2_. Data are presented as the mean ± SD of three independent experiments. ** *p* < 0.01, or *** *p* < 0.001 vs. control determined by one-way ANOVA.

Next, we tested whether HDAC8 depletion or inhibition would suppress the metastatic potential of melanoma. The Transwell assay and wound-healing assay results revealed that HDAC8 KO and PCI-34051 treatment greatly suppress the migration ability of melanoma cells (Figure 6A–D). Together, these results suggest that HDAC8 promotes cell proliferation and migration while validating the tumorigenic role of HDAC8 in melanoma progression.

### 3.5. HDAC8 and HIF-1α Expression Is Correlated with Poor Prognosis in Melanoma

**Figure 7:**
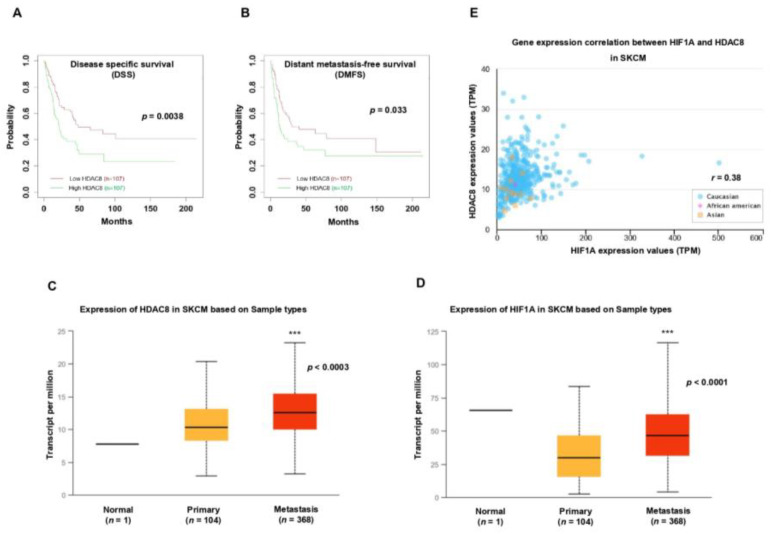
*HDAC8* and *HIF1A* expressions are correlated with poor prognosis in melanoma. (**A**,**B**) Kaplan–Meier survival analysis of patients with melanoma (*n* = 214). Patients were divided by *HDAC8* mRNA expression levels, and (**A**) their disease-specific survival (DSS) or (**B**) distant metastasis-free survival (DMFS) were assessed. Data were retrieved from the GSE65904 [27] database (*n* = 214). (**C**,**D**) Box-and-whisker plots displaying the expression of (**C**) *HDAC8* or (**D**) *HIF1A* in normal, primary, and metastatic melanoma samples. Data were analyzed using the UALCAN-based TCGA database (*n* = 473). (**E**) Correlation between *HIF1A* and *HDAC8* expressions in patients with melanoma. Data were analyzed using the UALCAN-based TCGA database (*n* = 473). *** *p* < 0.001 vs. control.

After revealing the crucial role of HDAC8 in regulating HIF-1α along with its tumorigenic function in melanoma in vitro, we analyzed the significance of *HDAC8* expression in melanoma patient survival. A Kaplan–Meier analysis of publicly available gene expressions from the GEO database (GSE65904 [36], *n* = 214) showed that high levels of *HDAC8* significantly correlated with poor disease-specific survival (DSS) (Figure 7A). Widespread metastases are the main cause of death in patients with melanoma [37]. Therefore, we analyzed the correlation of *HDAC8* expression with distant metastasis-free survival (DMFS). Patients with melanoma with high levels of *HDAC8* were associated with poor DMFS (Figure 7B). Next, we examined *HDAC8* and *HIF1A* expression patterns in normal, primary, and metastatic melanoma samples. Compared to primary melanoma, the expression levels of *HIF1A* and *HDAC8* were significantly elevated in metastatic melanoma (Figure 7C,D). Moreover, *HIF1A* expression was positively correlated with *HDAC8* expression in the samples from patients with melanoma (Figure 7E). Collectively, the patient survival analysis supports our findings that HDAC8 enhances the expression of HIF-1α and promotes tumor progression in melanoma.

## 4. Discussion

In this study, we delineated an epigenetic mechanism in which the epigenetic eraser HDAC8 stabilizes HIF-1α expression and promotes melanoma progression. HDAC8 interacts with HIF-1α and catalyzes its deacetylation, thereby promoting HIF-1α protein stability and transcriptional activity. Consequently, the depletion of HDAC8 suppresses cell proliferation and migration in melanoma. In patients with melanoma, *HDAC8* and *HIF1A* expressions show a positive correlation and the elevated expression of these genes is associated with poor prognosis. These data indicate HIF-1α as a novel HDAC8 substrate and suggest that HDAC8 may be an effective therapeutic target in melanoma.

Previous studies have identified several epigenetic regulators that interact with HIF-1α to regulate HIF-1α protein stability and transcriptional activity [32]. Although the Von Hippel–Lindau Tumor Suppressor (VHL) pathway is the major regulatory mechanism in determining the protein stability of HIF-1α, additional mechanisms have proven to affect the stability of HIF-1α under normoxic and hypoxic conditions. In particular, HIF-1α has been identified as the substrate of numerous HDACs and HATs. Depending on the HDAC and its specific deacetylation sites, HIF-1α deacetylation can either upregulate or deregulate its stability. For example, the deacetylation of lysine 709 (K709) by HDAC1 and the deacetylation of K674 by SIRT1 destabilize and inactivate HIF-1α [38,39]. However, the HDAC1/2-mediated deacetylation of K532 and the HDAC4-mediated deacetylation of the N-terminal region lead to the accumulation of HIF-1α [30,40,41]. The diverse ways in which HDACs can deacetylate HIF-1α reflect the complexity of HIF-1α regulation that remains to be further elucidated. In this study, we revealed a novel HDAC that is responsible for regulating HIF-1α expression. Through inhibitor screening, we identified that HDAC8 effectively regulates HIF-1α in melanoma cells. Further studies are required to verify the efficacy of the HDAC8-mediated deacetylation of HIF-1α in other cancer cell lines.

The tumorigenic role of HIF-1 is considerable. Various oncogenic pathways are activated by HIF-1. Not only does HIF-1 exert its effects on tumor cell proliferation, but it also affects apoptosis, angiogenesis, metastasis, immune evasion, and metabolism in cancer [42]. In melanoma, the hypoxic environment in the epidermis gives rise to the constitutive expression of HIF-1 and contributes to melanogenesis [11,43]. Furthermore, the expression and activity of HIF-1 are correlated with the malignant potential of melanoma cells and are considered important biomarkers for liver metastasis [26,44]. Accordingly, HIF-1 has been considered an effective therapeutic target for cancer treatment, and rigorous efforts have been devoted to developing HIF-1 inhibitors. However, due to the complexity of the regulation pathways, there have been no FDA-approved HIF-1 inhibitors thus far. Nevertheless, indirect HIF-1 inhibitors that target HIF-1 upstream pathways can lead to the inhibition of HIF activity. Since various HDACs are involved in promoting HIF-1α stability and activity, targeting HDACs can be a reasonable alternative for inhibiting HIF-1. Indeed, LBH589, a pan-histone deacetylase inhibitor, has been suggested as an indirect HIF inhibitor due to its influence on multiple HDACs, such as HDAC1, HDAC3, and HDAC4. These HDACs promote HIF-1α stability by directly binding to the oxygen-dependent degradation domain (ODDD) of HIF-1α [34]. However, pan-HDACi encompasses undesirable off-target toxicities. Therefore, targeting specific HDACs through selective inhibitors may be more appropriate for cancer treatment. Our results show that the HDAC8i PCI-30451 effectively suppresses HIF-1α, demonstrating it to be a novel indirect inhibitor of HIF-1α in melanoma.

HDAC8 plays a critical role in cancer development. It is involved in the enhancement of cell proliferation and metastasis, as well as in the suppression of apoptosis and evasion from the immune system. In melanoma, HDAC8 mediates the escape from BRAF inhibitor therapy, and HDAC8 and BRAF dual inhibition has been suggested as a potential therapeutic strategy for melanoma treatment [45]. In this study, we show that HDAC8 is an epigenetic regulator of HIF-1α, a critical transcription factor that drives tumor progression in various cancers, including melanoma. HDAC8 deacetylates and stabilizes HIF-1α, thereby promoting its transcriptional activity. Here, we demonstrate that a selective-HDAC8 inhibitor functions as an indirect inhibitor of HIF-1α and may be an effective therapeutic strategy in treating not only melanoma but also other solid tumors.

## 5. Conclusions

To conclude, we demonstrated the epigenetic regulation of HIF-1α in melanoma. HIF-1α is upregulated in melanoma and drives tumor progression. Therefore, targeting HIF-1α has been an attractive strategy in cancer treatment. However, various efforts concentrated on developing HIF-1α inhibitors have proven to be difficult. Here, we provide an alternative mechanism that regulates the expression and activity of HIF-1α. HDAC8 interacts with HIF-1α and mediates its deacetylation, enhancing the protein stability and transcriptional activity of HIF-1α. Importantly, targeting HDAC8 showed anti-cancer effects, such as suppressed cell proliferation and migration in melanoma. Therefore, HDAC8-selective inhibitors may function as indirect HIF-1α inhibitors and may be a promising strategy in treating patients with melanoma.

## Figures and Tables

**Table 1:** HDAC inhibitors and their selectivities.

Inhibitors	Selectivity	Concentration	Inhibitors	Selectivity	Concentration
Trichostatin A (TSA)	Pan-HDAC	2 µM	PCI-34051	HDAC8	20 µM
SAHA (Vorinostat)	Pan-HDAC	2 µM	TMP195	Class IIa HDAC	2 µM
LBH589 (Panobinostat)	Pan-HDAC	2 µM	TMP269	Class IIa HDAC	2 µM
Sodium Butyrate (NaB)	Pan-HDAC	2 µM	LMK-235	HDAC4/5	2 µM
FK228 (Romidepsin)	HDAC1/2	0.2 µM	ACY-241 (Citarinostat)	HDAC6	2 µM
T247	HDAC3	2 uM	Tenovin-6	SIRT1/2	10 µM

**Table 2:** The gRNA sequences targeting HDAC8.

Target	Oligonucleotide Sequences
gHDAC8 #1	F: 5′ CAC CGG CTG CCC AAT GCC TGA TTG A 3′ R: 5′ AAA CTC AAT CAG GCA TTG GGC AGC C 3′
gHDAC8 #2	F: 5′ CAC CGT AGG ATA GTT AAG CCT AAA G 3′ R: 5′ AAA CCT TTA GGC TTA ACT ATC CTA C 3′
gHDAC8 #3	F: 5′ CAC CGA TGC ACT GCA TAA GCA GAT G 3′ R: 5′ AAA CCA TCT GCT TAT GCA GTG CAT C 3′

**Table 3:** The qPCR primers used in this study.

Target Gene	Oligonucleotide Sequences
*HIF1A*	F: 5′ GAT TCG CCA TGG AGG GC 3′ R: 5′ TTC GAC GTT CAG AAC TCA TCT TTT 3′
*VEGFA*	F: 5′ TGA AGC CCT GGA GTG CGT 3′ R: 5′ AGG TTT GAT CCG CAT GAT CTG 3′
*SLC2A1*	F: 5′ TCT CTG TCG GCC TCT TTG TT 3′ R: 5′ GCA GAA GGG CAA CAG GAT AC 3′
*HK2*	F: 5′ AAG GCT TCA AGG CAT CTG 3′ R: 5′ CCA CAG GTC ATC ATA GTT CC 3′
*GAPDH*	F: 5′ CAT GAG AAG TAT GAC AAC AGC CT 3′ R: 5′ AGT CCT TCC ACG ATA CCA AAG T 3′

**Table 4:** Inhibition concentrations of PCI-34051 on cell growth and cell viability of melanoma cells.

Time	72 h		
	A2058	SK-MEL-2	SK-MEL-28
^1^ GI_50_ (µM)	15.38	14.39	17.62
^2^ IC_50_ (µM)	21.03	15.72	21.64

^1^ GI_50_: half-maximal growth inhibition concentration. ^2^ IC_50_: half-maximal cell viability inhibition concentration.

## Data Availability

The data presented in this study are available on request from the corresponding author.

## Supplementary Materials

The following supporting information can be downloaded at https://www.mdpi.com/article/10.3390/cancers15041123/s1, Figure S1: Transcriptional activity of HIF-1α is decreased upon PCI-34051 treatment; Figure S2: HDAC8 depletion suppresses cell proliferation in melanoma cells; Figure S3: Whole blot images of Figure 1A and Figure 1B; Figure S4: Whole blot images of Figure 2A–H; Figure S5: Whole blot images of Figure 3A–F; Figure S6: Whole blot images of Figure 4K,L.
