# Supplementary material for: HDAC8 Deacetylates HIF-1α and Enhances Its Protein Stability to Promote Tumor Growth and Migration in Melanoma
Source: Cancers (Basel). 2023 Feb 9;15(4):1123. doi: 10.3390/cancers15041123 (PMC9953989; doi:10.3390/cancers15041123)
Supplement: Supplementary file 1 [file cancers-15-01123-s001.zip › cancers-2171306-supplementary.pdf]

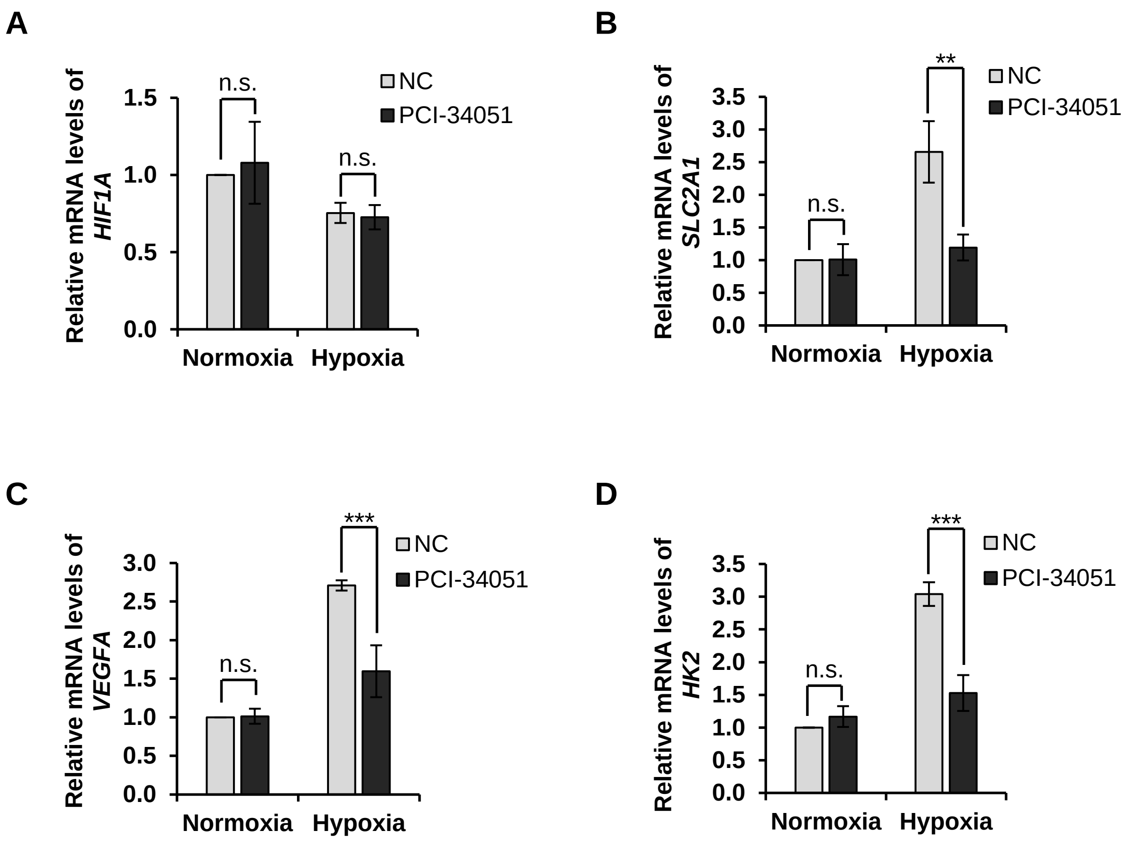

Figure S1. Transcriptional activity of HIF-1α is decreased upon PCI-34051 treatment. (**A-D**) qRT-PCR analysis of HIF-1α downstream target genes in A2058 cells treated with 0.1% DMSO or 20 μM PCI-34051 for 24 hours. Hypoxia was induced by incubating cells in 1% O_2. mRNA levels of (**A**) *HIF1A*, (**B**) *SLC2A1*, (**C**) *VEGFA*, and (**F**) *HK2* were compared to negative control (NC) upon PCI-34051 treatment under normoxia and hypoxia. mRNA levels of respective target genes were evaluated via qRT-PCR and semi-quantified relative to GAPDH gene expression. Data are presented as the mean ± SD of three independent experiments. ** $p < 0.01$, or *** $p < 0.001$ *vs*. control by one-way ANOVA. n.s., nonsignificant.

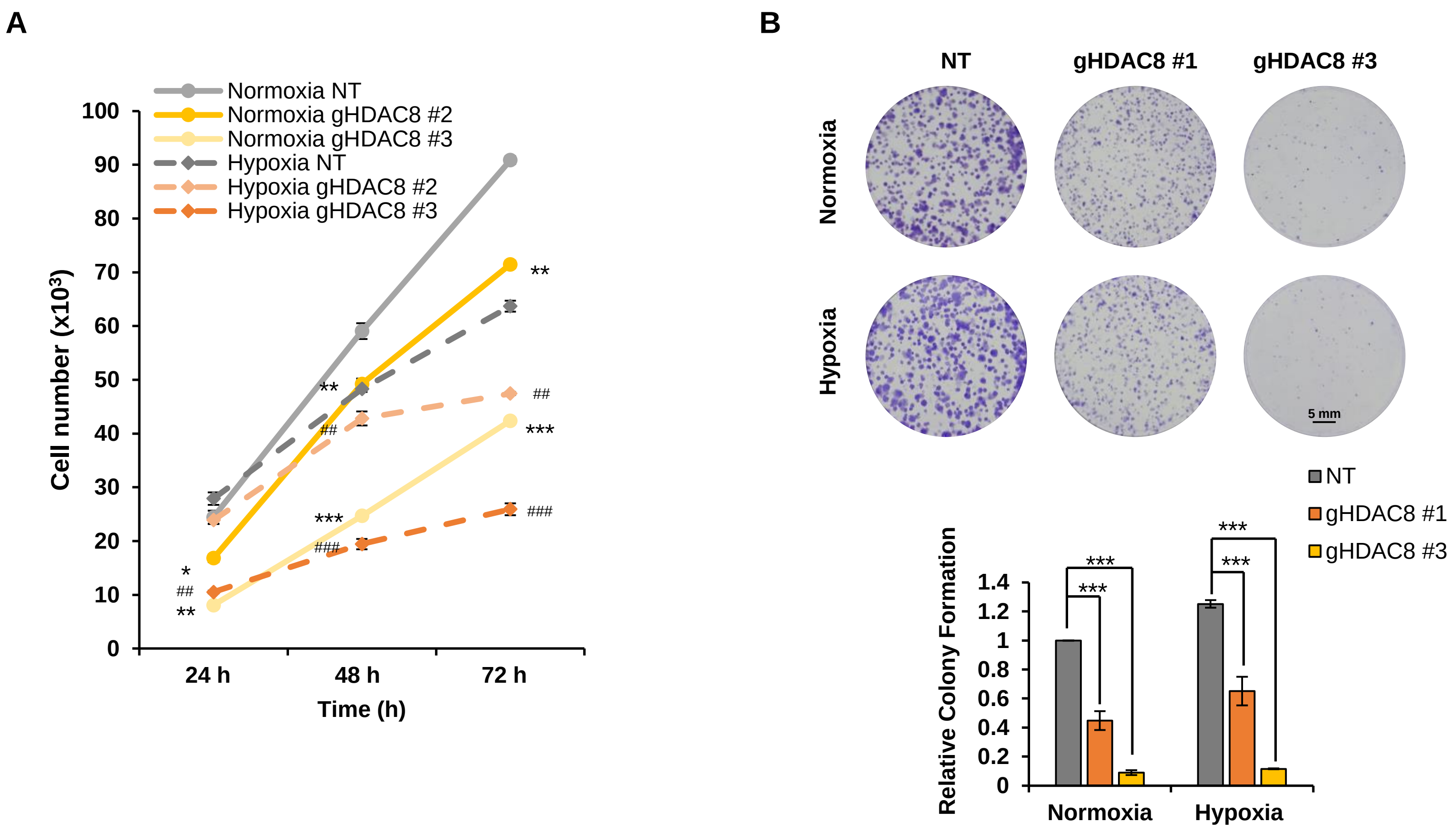

Figure S2. HDAC8 depletion suppresses cell proliferation in melanoma cells. (**A**) Cell growth curve of non-target (NT) control and gHDAC8 (HDAC8 KO) A2058 cells cultured for 24 h (h), 48 h, and 72 h under normoxic and hypoxic conditions. Cell growth was measured using the CCK-8 assay. Hypoxia was induced by incubating cells in 1% O_2. Data are presented as the mean ± SD of three independent experiments. * $p < 0.05$, ** $p < 0.01$, or *** $p < 0.001$ *vs*. normoxic control by Student's t-test. ## $p < 0.01$ or ### $p < 0.001$ vs. hypoxic control by Student's t-test. (**B**) Colony formation of NT and HDAC8 KO A2058 cells were incubated for 12 days under normoxic and hypoxic conditions. Scale bar = 5 mm. Hypoxia was induced by incubating cells in 1% O_2. Data are presented as the mean ± SD of three independent experiments. *** $p < 0.001$ *vs*. control by one-way ANOVA.

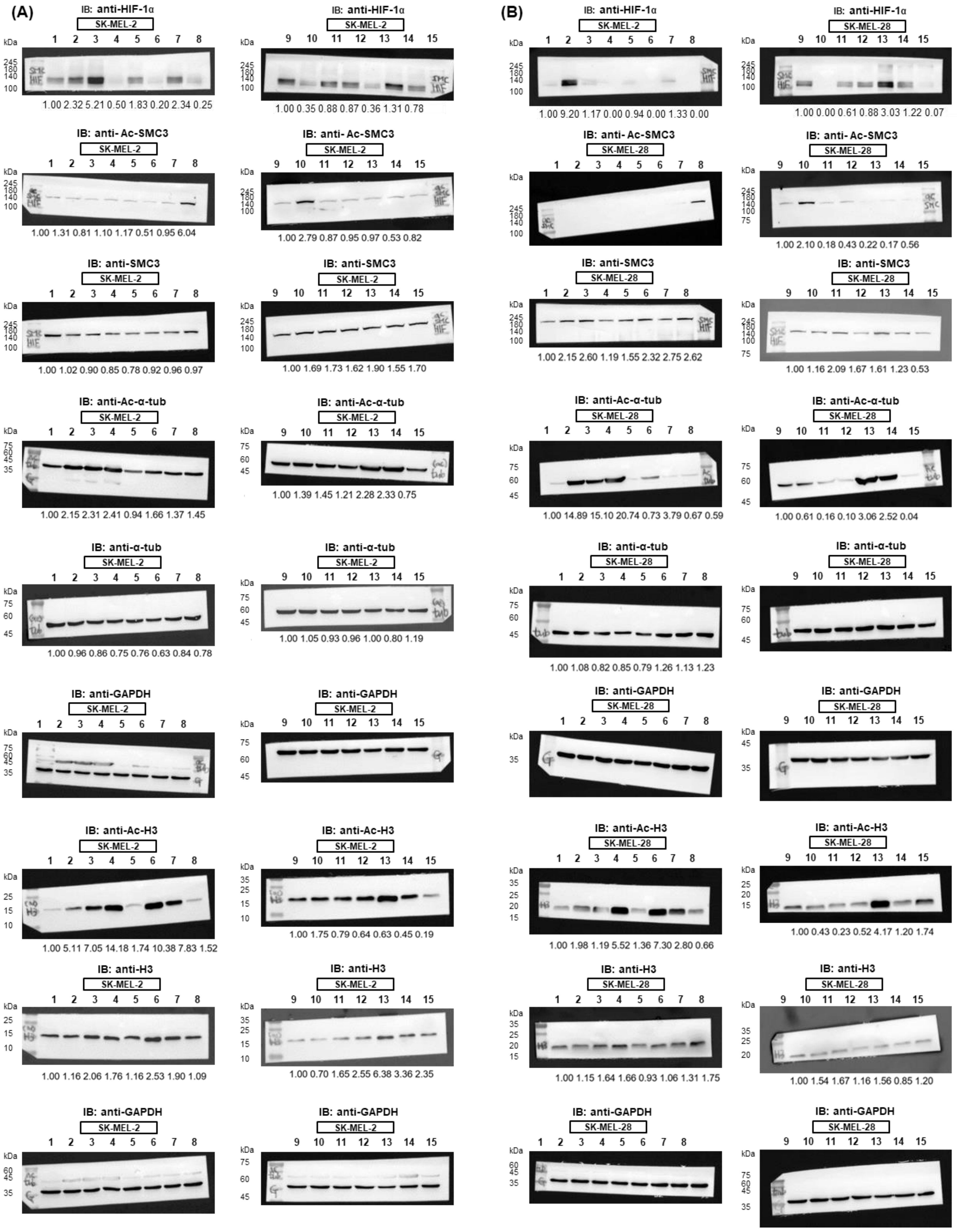

Figure S3. Whole blot images of Figure 1A and Figure 1B.

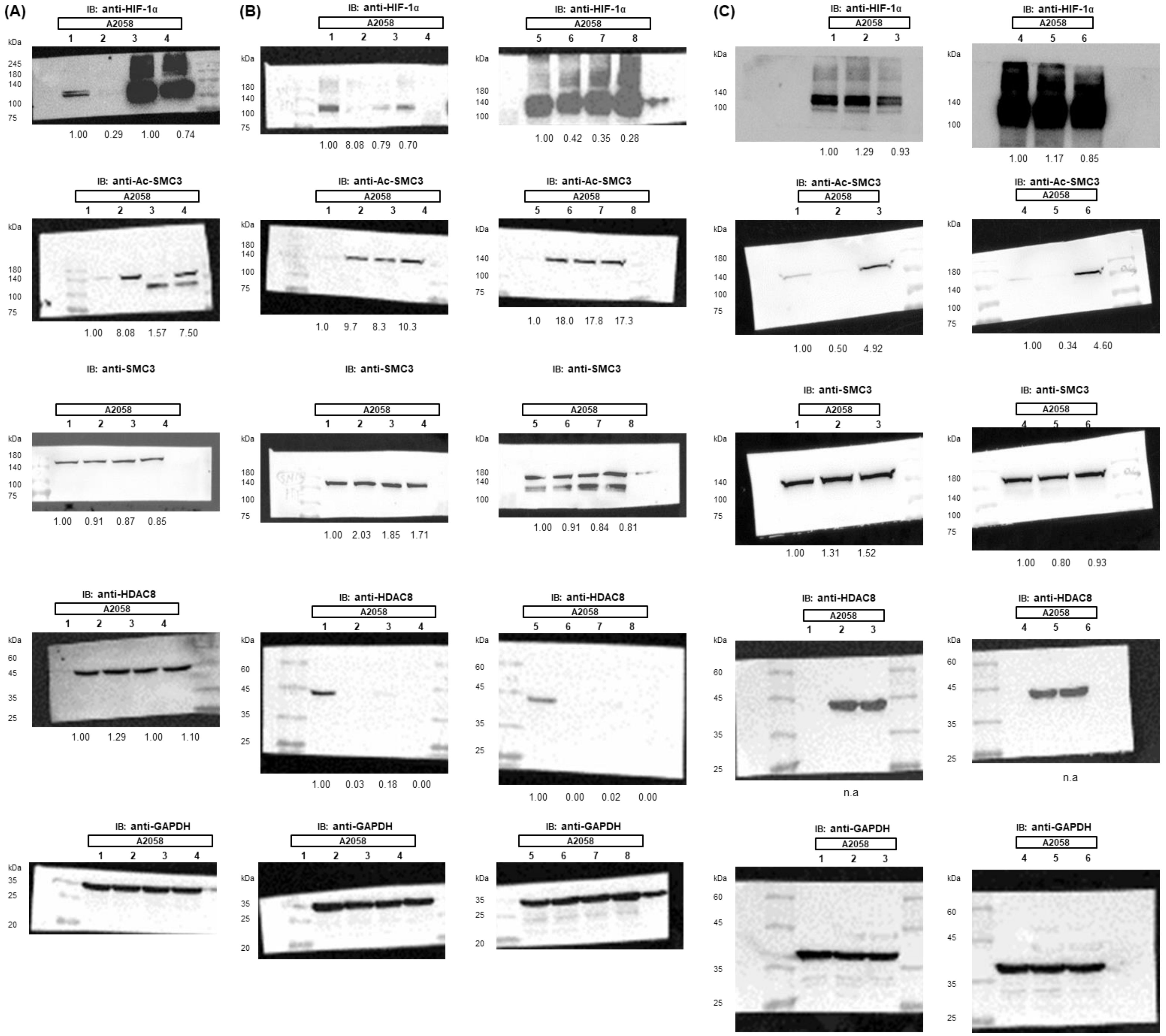

Figure S4. Whole blot images of Figure 2A – Figure 2H.

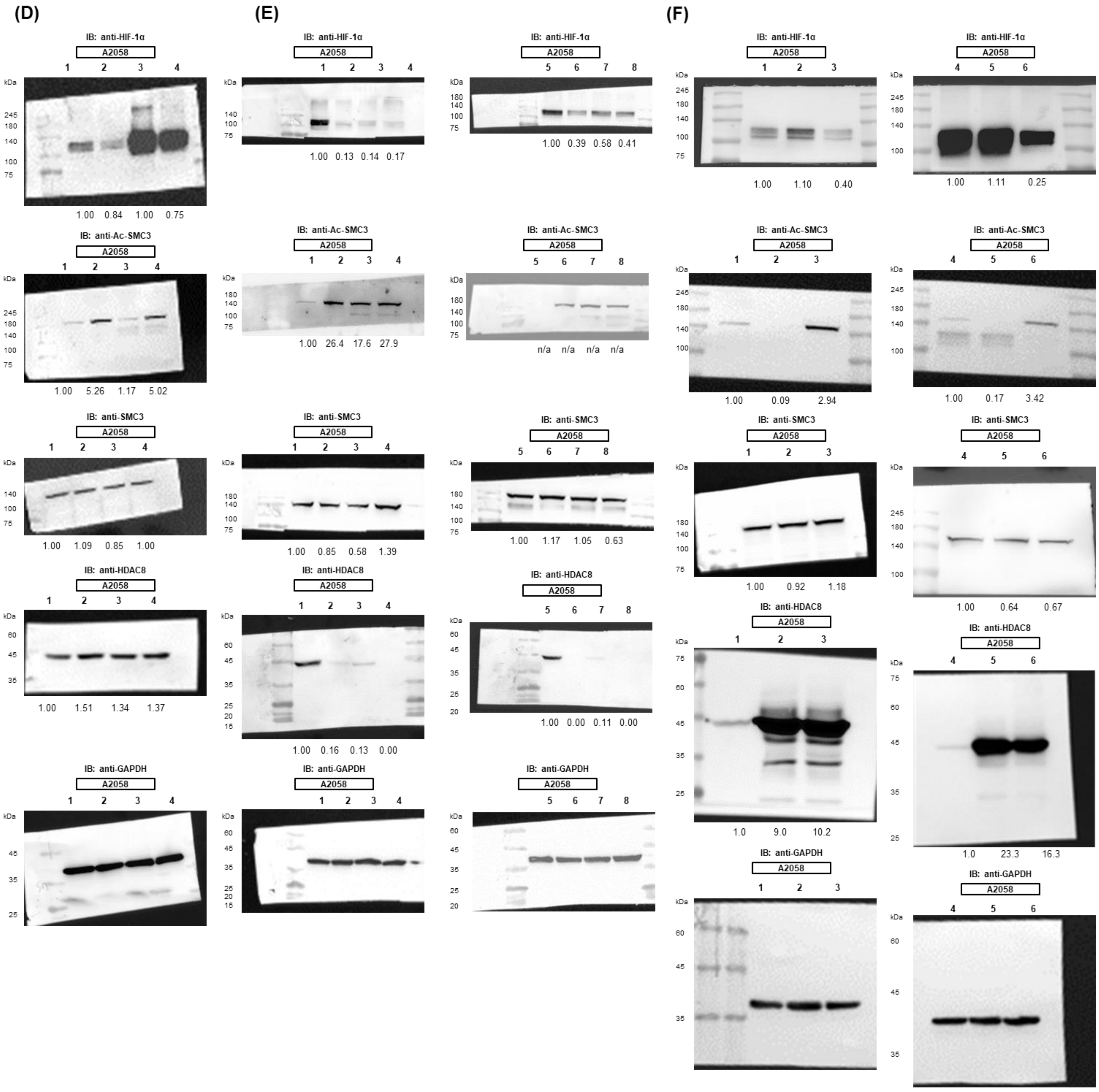

Figure S4. Whole blot images of Figure 2A – Figure 2H.

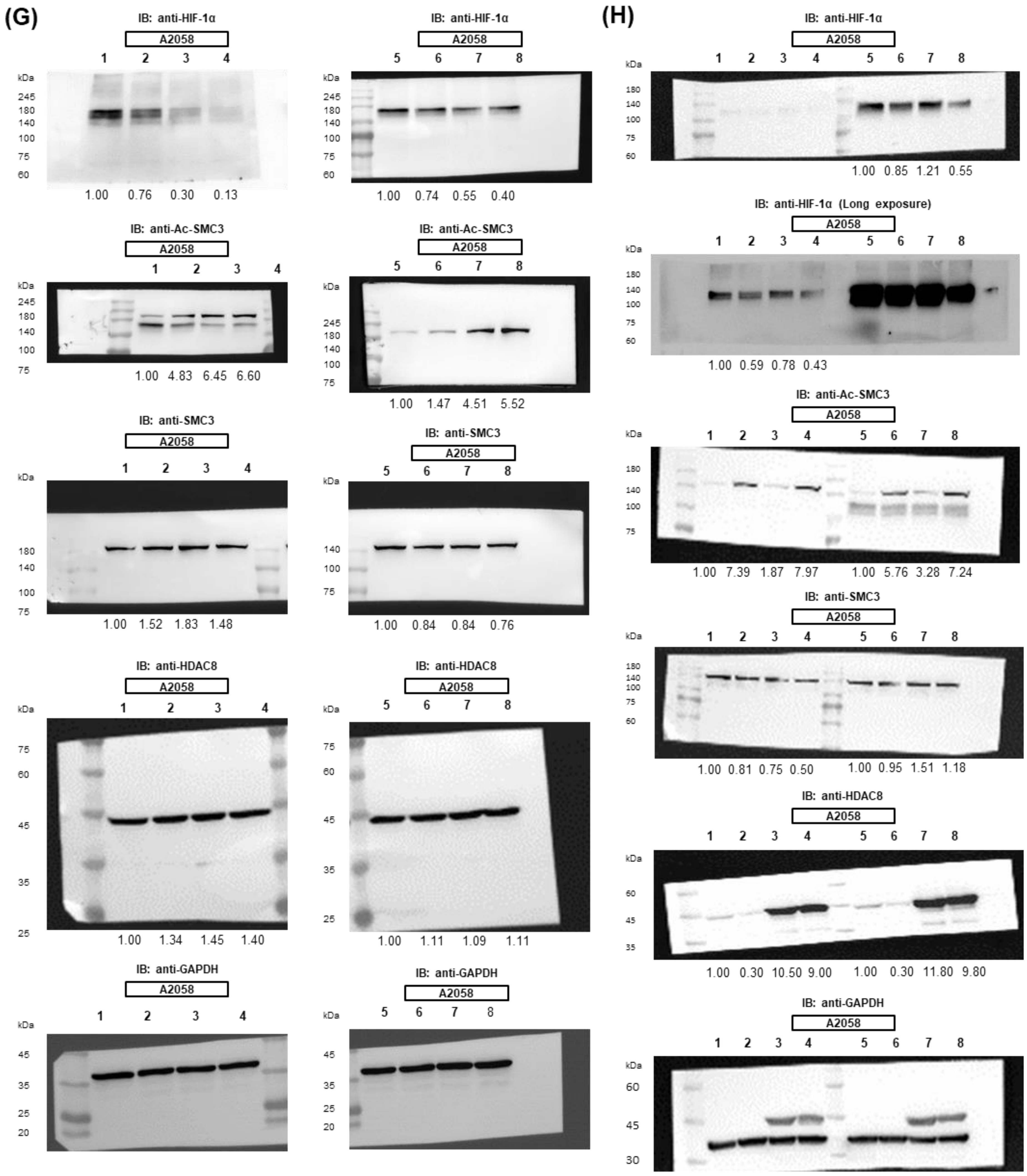

Figure S4. Whole blot images of Figure 2A – Figure 2H.

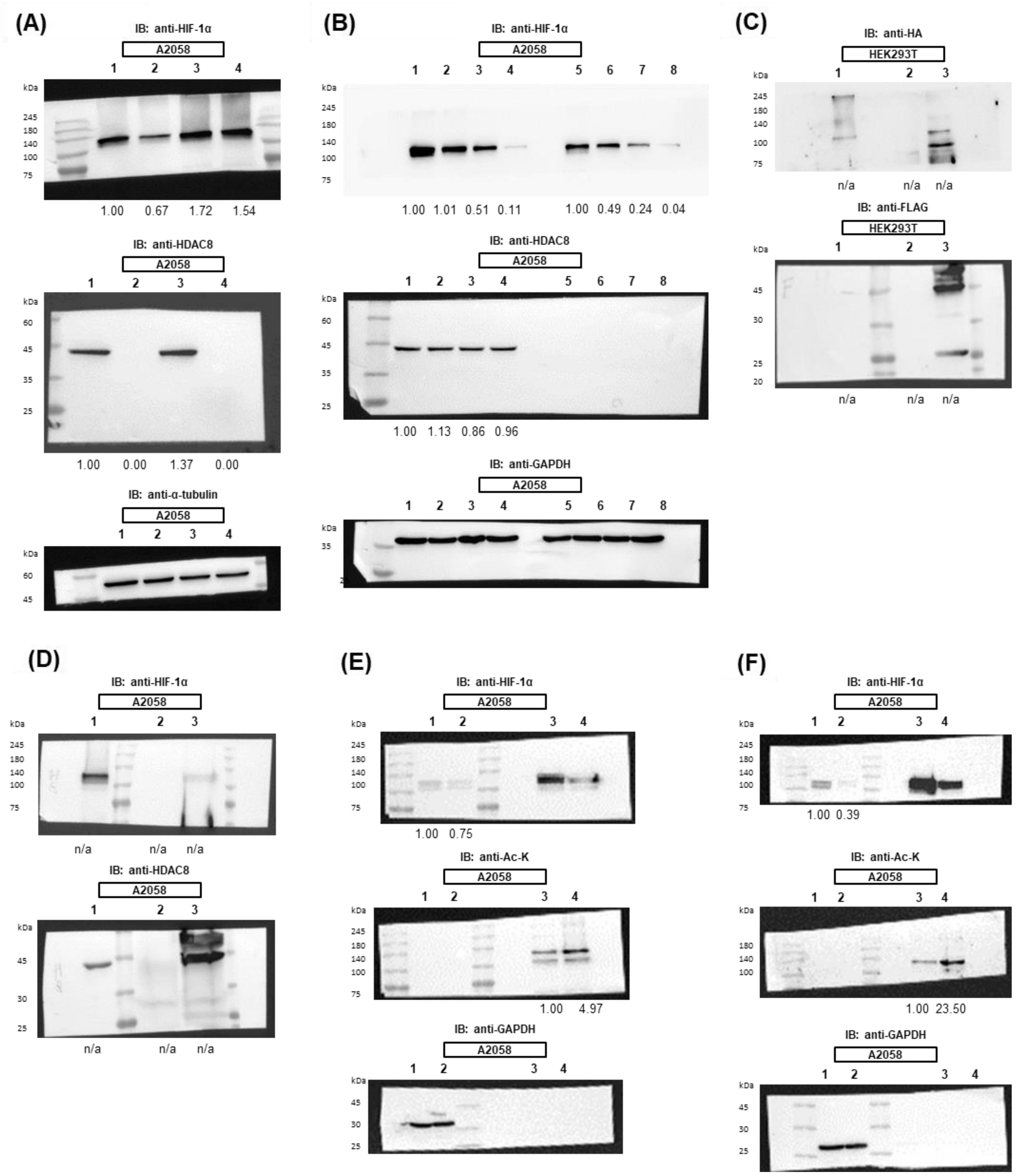

Figure S5. Whole blot images of Figure 3A - Figure 3F.

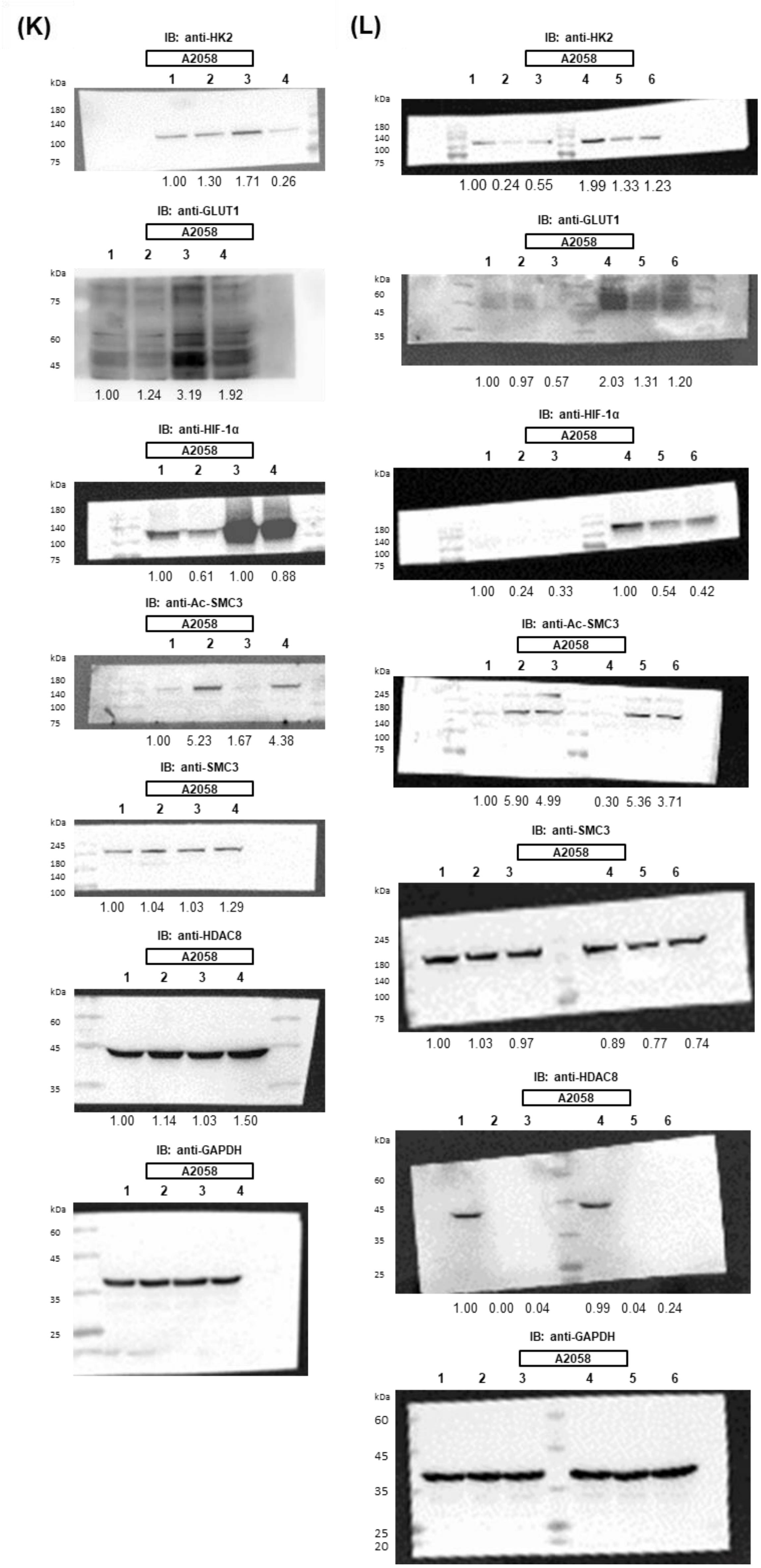

Figure S6. Whole blot images of Figure 4K and Figure 4L.
